# Impact of chronic sexual abuse and depression on inflammation and wound healing in the female reproductive tract of HIV-uninfected and HIV-infected women

**DOI:** 10.1371/journal.pone.0198412

**Published:** 2018-06-12

**Authors:** Mimi Ghosh, Jason Daniels, Maria Pyra, Monika Juzumaite, Mariel Jais, Kerry Murphy, Tonya N. Taylor, Seble Kassaye, Lorie Benning, Mardge Cohen, Kathleen Weber

**Affiliations:** 1 Department of Epidemiology and Biostatistics, Milken Institute School of Public Health, George Washington University, Washington DC, United States of America; 2 Department of Epidemiology, School of Public Health, University of Washington, Seattle, WA, United States of America; 3 Albert Einstein College of Medicine/Montefiore Medical Center, Bronx, NY, United States of America; 4 SUNY Downstate Medical Center, Brooklyn, NY, United States of America; 5 Georgetown University Medical Center, Washington DC, United States of America; 6 Department of Epidemiology, Johns Hopkins Bloomberg School of Public Health, Baltimore, MD, United States of America; 7 Department of Medicine, John H. Stroger Jr Hospital of Cook County, Chicago, IL, United States of America; 8 Cook County Health and Hospitals System/ Hektoen Institute of Medicine, Chicago, IL, United States of America; Stellenbosch University, SOUTH AFRICA

## Abstract

Sexual violence is associated with increased risk of HIV acquisition/transmission in women. Forced sex can result in physical trauma to the reproductive tract as well as severe psychological distress. However, immuno-biological mechanisms linking sexual violence and HIV susceptibility are incompletely understood. Using the Women’s Interagency HIV Study repository, a total of 77 women were selected to form 4 groups, stratified by HIV serostatus, in the following categories: 1) no sexual abuse history and low depressive symptom score (below clinically significant cut-off, scores <16) (Control); 2) no sexual abuse history but high depressive symptom score, ≥16 (Depression); 3) chronic sexual abuse exposure and low depressive symptom score (Abuse); 4) chronic sexual abuse exposure and high depressive symptom score (Abuse+Depression). Inflammation-associated cytokines/chemokines/proteases (TNF-α, IL-6, IL-1α, IL-1β, TGF-β MIP-3α, IP-10, MCP-1, Cathepsin B), anti-inflammatory/anti-HIV mediators (Secretory leukocyte protease inhibitor (SLPI), Elafin, beta defensin 2 (HBD2), alpha defensins (HNP 1–3), Thrombospondin (TSP-1), Serpin A1, A5, Cystatin A, B), and wound-healing mediators (Gro-α, VEGF, PDGF, EGF, FGF, IGF), were measured in cervical-vaginal lavage (CVL) using ELISA. Linear regression was used to model association of biomarkers with depression and abuse as predictor variables; the interaction between depression and abuse was also tested. Anti-HIV activity in CVL was tested using TZM-bl indicator cell line. In HIV-uninfected women, median levels of IL-6 (p = 0.04), IL-1α (p<0.01), TGF-β (p = 0.01), IP-10 (p = <0.01), PDGF (p<0.01) and FGF (p<0.01), differed significantly between groups. Specifically, an association was found between chronic sexual abuse and increased IL-1α (p<0.01), MIP-3α (p = 0.04), IP-10 (p<0.01), Serpin B1 (p = 0.01), FGF (p = 0.04) and decreased TGF-β (p<0.01), MCP-1 (p = 0.02), PDGF (p<0.01). Further, there was evidence of significant interactions between chronic sexual abuse and current depression for IL-1α, IP-10, Serpin A1, Cystatin B, and FGF. In HIV-infected women, median levels of TNF-α (p<0.01), IL-6 (p = 0.05), MIP-3α (p<0.01), and MCP-1 (p = 0.01), differed significantly between groups. Specifically, an association was found between chronic sexual abuse and increased MCP-1 (p = 0.03), Gro-α (p = 0.01) and decreased TNF-α (p<0.01), IL-1α (p = 0.02), MIP-3α (p<0.01) and Cathepsin B (p = 0.03). Current depressive symptoms were associated with significantly decreased MIP-3α (p<0.01). There was evidence of significant interactions between chronic sexual abuse and current depression for MCP-1 and FGF. No significant differences were observed in anti-HIV activity among all eight groups. Heat-map analyses revealed distinct immune network patterns, particularly in the Abuse groups for both HIV-infected and uninfected women. Our data indicates a complex relationship between chronic sexual abuse exposure, depressive symptoms, and FRT immune mediators that are also affected by HIV status. Association of chronic sexual abuse with increase in inflammation-associated cytokine/chemokine expression, along with impaired wound-healing associated growth-factors can create a microenvironment that can facilitate HIV infection. Evaluation of longitudinal changes in exposures and biomarkers are needed to untangle the immuno-biological mechanisms that may put women who endure life-long sexual abuse at increased risk for HIV.

## Introduction

Violence and HIV/AIDS form a tragic feedback loop that disproportionately affects women worldwide [1]. In the United States alone, partner violence and sexual assault are the first and sixth leading causes of injury in women, respectively [2],[3]. Globally, it is estimated that about 1 in 3 women experience physical violence in their lifetimes, most of which is committed by an intimate partner [4]. Women are also more likely to be affected by the HIV/AIDS epidemic. As of 2015, 51% of all HIV infections were in women, with the greatest burden (60%) among young adults [5]. Violence against women has been significantly associated with increased risk of HIV acquisition/transmission [1],[6].

Heterosexual sex is the most common route of HIV infection in women [7]. Several attributes of the female reproductive tract (FRT) can modulate susceptibility to HIV infection, including the presence of the sex hormones estrogen and progesterone. The sex hormones modulate sexually transmitted infection (STI) susceptibility throughout the menstrual cycle by affecting the mucosal environment within the FRT [8], [9]. Recent studies have identified the secretory phase as being a window of increased HIV susceptibility [9]. Susceptibility to infection is also increased by the presence of cervico-vaginal lymphocytes (which can be activated in response to injury) and dysregulation of epithelial tight junctions within the FRT (which results in increased permeability and increased access for pathogens) [8],[9].

The impact of sexual violence on the FRT is of particular interest to HIV acquisition/transmission because most sexual assaults involve vaginal penetration [3]. There are many physical changes that occur within the FRT after sexual assault that can be associated with increased risk of HIV transmission, including vaginal lacerations, abrasions, and inflammation [3],[10]. Alterations to T-cell function and slower wound healing have also been reported in survivors of violence, both of which are factors that increase susceptibility to HIV infection [6]. Wounds resulting in damaged mucosal surfaces are estimated to occur in 22–90% of all sexual assaults [3].

Wound healing is a dynamic process that is tightly regulated by the timed sequential release of cytokines, chemokines, proteases/anti-proteases and growth factors by platelets, macrophages, neutrophils, and fibroblasts [11],[12]. The process begins when inflammation occurs as a result of mucosal trauma and injury (as from sexual assault). The repair process begins immediately with sequential release of specific immune factors that act to control inflammation and recruit the cells necessary for repair. This is followed by new tissue formation and remodeling, which can take weeks to months [11],[12]. Improper/untimely expression of any of these mediators can result in delayed wound healing and increased susceptibility to pathogens such as HIV [12][13]. Expression of soluble immune mediators involved in the wound healing process can be affected by factors such as the presence of infections, history of chronic injury, age and stress, as well as behavioral variables such as smoking and alcohol consumption [14]. Mucosal wound healing is also gender-specific with significantly delayed healing reported in women compared to men [15] [16]. Further, many of the mediators are regulated by sex hormones and hence vary throughout the menstrual cycle [17]. The anti-inflammatory/anti-proteases such as SLPI [18] and Elafin [19], which are critical for timely control of the initial inflammatory response, also have direct HIV inhibitory functions [20]. Whereas it is known that HIV-infected individuals experience chronic inflammatory conditions even while on antiretroviral therapy [21], studies are limited on how wound healing pathways are affected in HIV-infected women, particularly those exposed to sexual violence.

In addition to the physical effects of violence, those exposed to it are more likely to engage in high-risk sexual behaviors. For example, intimate partner violence (IPV) victims are more likely to have transactional and unprotected sex, and are less likely to be tested for HIV [1],[6]. Women in abusive relationships are at increased risk of contracting an STI as well as suffer from depression, than women that are in relationships that are abuse free. [6],[22]. HIV status is also associated with IPV, with HIV-infected women in the United States experiencing double the rate of IPV as HIV-uninfected women [6]. Further, depression is a common occurrence in women who have experienced violence [23], and depression is known to negatively impact the immune system [24]. A recent meta-analysis that included 37 studies [23] found 2-3-fold increased risk of major depressive disorder in women exposed to intimate partner violence (IPV), particularly in those experiencing life-time chronic exposure. In terms of immune dysregulation, depression has been associated with both immune activation (increase in pro-inflammatory mediators such as IL-6, TNF-α, CRP, IL1) and immune suppression (loss of T cell and NK cell function) (reviewed [25]). Pro-inflammatory cytokines endogenously produced in the brain can directly impact depressive symptoms by affecting neuronal growth and plasticity [26] and a handful of studies have reported anti-cytokine therapy to effectively reduce depressive symptoms [27]. In addition, certain polymorphisms in TNF-α, which can directly activate serotonin pathways in the brain, have been linked to major depressive illnesses [28]. Depressive symptoms have also been associated with dysregulation of HPA axis and cortisol mediated stress response (reviewed [29]). Finally, there is some evidence of association between depressive symptoms and significantly slower rates of mucosal wound healing [30]. Although causality of inflammation/immune dysregulation and depression are difficult to prove, a study by Das [31] found inflammation to be a predictor of psychosocial distress.

Overall, there is limited data available to help us with our understanding of the specific immuno-biological mechanisms associated with sexual abuse exposure, depressive symptoms, and HIV status, individually and in combination. In this study, we sought to identify differences in FRT immune mediators by HIV status based on sexual abuse histories and current depressive symptoms. We hypothesized that women with the greatest burden of chronic sexual abuse and current depressive symptoms would have a genital immune dysregulation pattern of high inflammation/immune activation and impaired wound healing pathways which can negatively impact risk of HIV acquisition/transmission.

## Materials and methods

### Ethical statement

The Women’s Interagency HIV Study (WIHS) protocol and this study were conducted according to the principles expressed in the Declaration of Helsinki. After approval by each participating institution’s review board, study staff obtained written informed consent for the collection and use of data and specimens from each research participant. All participants consented to future use of their data and their specimens in the repository. This study was determined to not meet the definition of human subjects research by The George Washington University IRB, as all our analyses involved de-identified samples without access to any code-link.

### Study participants and demographics

WIHS is an ongoing prospective observational cohort study of HIV-infected and sociodemographically similar uninfected women in the United States. Study methods, baseline cohort characteristics, and long-term retention have been previously described [32–34]. Briefly, semiannual visits included an in-depth interview for collection of demographic, behavioral, and clinical factors, a physical and gynecologic exam with specimen collection for the repository. For this cross-sectional study, participants were selected from either the 1994–95 or 2001–02 WIHS enrollment periods based on the following criteria: complete baseline and longitudinal childhood and adult abuse data, as well as, not pregnant at visit, breast feeding, or lacking a cervix as a result of hysterectomy. Individuals selected based on these criteria were then categorized based on their self-reported sexual abuse histories.

In both HIV-infected and uninfected women, we identified sexual abuse groups representing life-long history of multiple types of sexual abuse exposure (childhood and adult sexual abuse including any reported transactional sex), which we defined as “chronic sexual abuse”. Comparison groups (Controls) had never been exposed to sexual abuse. From among visits with cervicovaginal lavage (CVL) aliquots available in the repository, we selected a visit associated with concurrent sexual abuse exposure. We then further stratified visits based on the level of concurrent self-reported depressive symptoms using the Center for Epidemiologic Studies-Depression (CES-D) [35]) Scale. This is a measure that asks questions about symptoms associated with depression. Scores range from 0 to 60, with high scores indicating greater depressive symptoms; a score of >16 is used as a cut-off for clinical depression ([36], [37]).

Visits from a total of 77 women were selected to form 4 groups stratified by HIV serostatus in the following categories: 1) no sexual abuse history and low depressive symptom score (below clinically significant cut-off, scores <16) (Control); 2) no sexual abuse history but high depressive symptom score, ≥16 (Depression); 3) chronic sexual abuse exposure and low depressive symptom score (Abuse); 4) chronic sexual abuse exposure and high depressive symptom score (Abuse+Depression). Plasma viral load and CD4 counts in HIV-infected women, were obtained from WIHS database. CVL viral loads were also quantified; however, almost 100% of the samples were undetectable at a detection level of 40 copies/mL. All HIV-infected women were on highly active antiretroviral therapy (HAART).

### Measurement of cytokines, chemokines, and antimicrobials in cervico-vaginal lavage

CVL samples were obtained from WIHS repository and stored at –80° C until assayed for cytokines: TNF-α, IL-6, IL-1α, IL-1β, TGF-β; chemokines: MIP-3α, IL-8, MCP-1, IP-10; antimicrobial/antiproteases: secretory leukocyte protease inhibitor (SLPI), Elafin, Human beta defensin 2 (HBD2), Human alpha defensins 1–3 (HNP 1–3), Thrombospondin (TSP-1), Serpin A1, Serpin A5, Cystatin A, Cystatin B; growth factors/proteases: Gro-α, vascular endothelial growth factor (VEGF), platelet derived growth factor (PDGF), epidermal growth factor (EGF), fibroblast growth factor (FGF), insulin-like growth factor (IGF), and Cathepsin B with ELISA Quantikine or Duoset kits. All except HBD-2, Serpin A1, B1 and Cystatin A, were purchased from R&D Systems (Minneapolis, MN), and assays were performed according to the manufacturer’s protocols. HBD2 was assayed using an ELISA test kit from PeproTech (Rocky Hill, NJ). Serpin A1, B1 and Cystatin A ELISA kits were purchased from Lifespan Biosciences (Seattle, Washington). All immune mediators were quantified based on standard curves obtained using a Microplate Reader (Biotek, Winooski, VT). Biomarker concentrations below the lower limit of detection were reported as the mid-point between the lowest concentrations measured and zero.

### Total protein analysis

Total protein concentration in CVL samples was determined using the Pierce BCA protein assay kit (Thermo-Fisher Scientific), following manufacturer’s instructions.

### HIV viral stocks

Laboratory-adapted HIV-1 strain BaL was kindly provided by Dr. P. Gupta (University of Pittsburgh, PA). We also used HIV-1 transmitted/founder (T/F) strain RHPA.c, a Clade B infectious molecular clone (IMC)[38] matching the inferred T/F virus nucleotide sequence from subject RHPA4259 and isolated from a heterosexually-infected female subject (kindly provided by Dr. Christina Ochsenbauer, University of Alabama at Birmingham, AL). Virus stocks were propagated in PHA-stimulated human PBMC and stored frozen at −80° C. Virus titers were determined on TZM-bl cells.

### HIV inhibition analysis

Anti-HIV activity in CVL was determined using TZM-bl indicator cell-line (AIDS reagent Repository, NIH), essentially as previously described [39]. Cells were seeded at 2×10^4^ cells per well in a 96-well plate, and allowed to adhere overnight at 37° C. CVL samples were diluted 1:4 in TZM-bl media (phenol red-free DMEM (Invitrogen Life Technologies, Carlsbad, CA) supplemented with 10% defined FBS (HyClone, Logan, UT), 2 mM L-glutamine (Invitrogen Life Technologies), and 50 μg/mL Primocin (Invivogen, San Diego, CA) and incubated with a laboratory-adapted HIV-1 strain BaL, as well as a heterosexually transmitted (T/F) strain RHPA.c (at 250 tissue culture infectious dose (TCID_50_). The dose was selected to minimize virus-induced cytopathic effects, while maintaining an ability to measure >1 log reduction in virus infectivity (standardized by [40]). Samples were incubated with virus for 1 hour at 37° C and added to TZM-bl cells. Luciferase activity was measured after 48h upon application of substrate beta-Glo (Promega, Madison, WI). Uninfected cells and cells treated with CVL alone were used to determine background luminescence expressed as relative light units (RLU). All conditions were tested in triplicates and repeated twice. To calculate percent inhibition, RLU values of “virus only” wells were averaged and set to 100%. Viability of cells upon treatment with CVL was quantified using the CellTiter 96® AQueous One Solution Cell Proliferation Assay (Promega) according to manufacturer’s instructions. Briefly, reagent was added directly to cell cultures and incubated for 30 min at 37° C followed by reading the plate in a plate reader at OD 490 nm.

### Statistical analyses

Possible covariates that can affect immune biomarkers in the context of our study of interest were compared across the four groups, separately by HIV status; chi-square or Fishers exact test was used for categorical variables, and the Kruskal-Wallis test for continuous variables. All biomarker level values were log-transformed as they were not normally distributed. The median log-transformed values of each biomarker were compared by group, using the Kruskal-Wallis test. For biomarkers with significant differences in medians, graphs were created using R Studio version 1.0.136 with Mann-Whitney U tests comparing each group to the control.

Heat-maps for each group were constructed to assess the direction and strength of associations between individual biomarkers and other functional and clinical variables such as HIV inhibition, CD4, and plasma viral load data by performing Spearman’s rank-order correlation tests using R Studio version 1.0.136.

We ran linear regression models, separately for HIV- uninfected and HIV-infected women, for each immune mediator. For HIV-uninfected women, we ran unadjusted analyses with both abuse and depression as predictors. Due to the small sample size, we did not adjust models for hormonal contraception or Bacterial Vaginosis (BV), covariates which can be associated with biological changes in the FRT [41,42].

Models for HIV-infected women were adjusted for CD4+ T cell count and plasma HIV viral load. Final models included an interaction term between abuse and depression.

Models including all women were used to assess effect modification by HIV status. For each biomarker, the predictors included abuse, depression, HIV status and two interaction terms: HIV status and abuse, and HIV status and depression. These terms tested whether the difference between women who reported chronic sexual abuse versus no abuse (or reported depressive symptoms vs no symptoms) differed by HIV status. Given the large numbers of models and outcomes tested, we highlight results that remained significant after using a Bonferroni correction. These analyses were conducted in SAS 9.4.

## Results

### Characteristics of study samples

Among HIV-uninfected women (Table 1A), women in the Control group were younger (p<0.01) and in the higher income category (p<0.001). Women who were not Controls were more likely to be current smokers (p<0.01), report substance use (p<0.01) and consume more alcoholic drinks per week (Abuse+Depression group) (p<0.01), compared to the other groups. Women in the Abuse+Depression group were more likely to have reported transactional sex since last visit (p = 0.03). Women also differed significantly in terms of any abuse history (P<0.01), any abuse since last visit (p<0.01), childhood sexual and physical abuse (p<0.01) and adult physical abuse history (P<0.01), in addition to the selection criteria variables of current depression and sexual abuse exposures (p<0.01).

**Table 1:** Participant characteristics among (A) HIV-uninfected and (B) HIV-infected women. Any Abuse history includes emotional, physical, sexual abuse. SLV: Since last visit, in the past 6 months. Some categories have missing values and therefore, might not add up to the sample size for that group.

**1A. HIV-uninfected**					
**Variable**	**No Depression, No Abuse, %(N)**	**Depression Only, %(N)**	**Abuse Only, %(N)**	**Depression & Abuse, %(N)**	**p-value**
N	10	11	10	8	
**Demographics**					
Age, Median (IQR)	35.2 (30.6–39.5)	47.9 (46.8–48.8)	41.0 (39.4–49.5)	41.8 (34.5–44.1)	**<0.01**
Race					0.15
White	20.0 (2)	36.4 (4)	50.0 (5)	12.5 (1)	.
African-American	80.0 (8)	36.4 (3)	40.0 (4)	75.0 (6)	.
Other	0.0 (0)	27.3 (3)	10.0 (1)	12.5 (1)	.
Education					0.39
<HS	10.0 (1)	54.5 (5)	11.1 (1)	37.5 (3)	.
HS	20.0 (2)	18.2 (2)	22.2 (2)	25.0 (2)	.
>HS	70.0 (7)	27.3 (3)	66.7 (6)	37.5 (3)	.
Income					**<0.01**
<$12,000	0.0 (0)	60.0 (6)	44.4 (4)	50.0 (3)	.
<$24,000	0.0 (0)	20.0 (2)	11.1 (1)	50.0 (3)	.
>$24,000	100.0 (10)	20.0 (2)	44.4 (4)	0.0 (0)	.
Employed	80.0 (8)	27.3 (2)	33.3 (3)	37.5 (3)	0.07
**Reproductive status and sexual behavior**					
					
# Male Sex Partners (last 6 months), Median (IQR)	1.0 (1.0–1.0)	1.0 (1.0–1.0)	1.0 (1.0–1.0)	1.0 (1.0–5.0)	0.22
Vaginal Sex with Male last 48 hrs	20.0 (2)	9.1 (1)	11.1 (1)	25.0 (2)	0.82
Condom Use During Vaginal Sex					0.54
Always	33.3 (3)	44.4 (4)	12.5 (1)	0.0 (8)	.
Sometimes	33.3 (3)	11.1 (1)	37.5 (3)	28.6 (2)	.
Never	33.3 (3)	44.4 (4)	50.0 (4)	71.4 (5)	.
Anal Sex SLV	0.0 (0)	0.0 (0)	12.5 (1)	0.0 (0)	0.7
Transactional Sex SLV	0.0 (0)	0.0 (0)	0.0 (0)	25.0 (2)	**0.03**
BV by Amsel at visit	12.5 (1)	12.5 (1)	10.0 (1)	0.0 (0)	1
Been Through Menopause	0.0 (0)	27.3 (3)	22.2 (2)	12.5 (1)	0.31
Hormonal Contraception last 6 months	10.0 (1)	18.2 (2)	10.0 (1)	0.0 (0)	0.89
**Other risk behavior and abuse/depression status**					
Current Smoker	10.0 (1)	81.8 (9)	22.2 (2)	50.0 (4)	**<0.01**
Ever use crack, cocaine, or heroin	10.0 (1)	90.9 (10)	66.7 (6)	75.0 (6)	**<0.01**
Drinks per week, Median (IQR)	0.7 (0.1–1.5)	0.0 (0.0–5.5)	0.0 (0.0–0.1)	12.3 (4.3–26)	**<0.01**
CESD, Median (IQR)	0.0 (0.0–0.0)	42.0 (32.0–44.0)	6.5 (5.0–10.0)	26.5 (23.5–33.0)	**<0.01**
Any Abuse history	40.0 (4)	81.8 (8)	100 (9)	100 (8)	**<0.01**
Any Abuse SLV	0.0 (0)	10.0 (1)	22.2 (2)	62.5 (5)	**<0.01**
Childhood Sexual Abuse	0.0 (0)	0.0 (0)	100 (10)	100 (8)	**<0.01**
Childhood Physical Abuse	0.0 (0)	18.2 (2)	66.7 (6)	37.5 (3)	**<0.01**
IPV history	10.0 (1)	36.4 (4)	66.7 (6)	50.0 (4)	0.07
IPV SLV	0.0 (0)	9.1 (1)	11.1 (1)	37.5 (3)	0.12
Sexual Abuse history	0.0 (0)	0.0 (0)	100 (10)	100 (8)	**<0.01**
Sexual Abuse SLV	0.0 (0)	0.0 (0)	0.0 (0)	25.0 (2)	.
Physical Abuse history	30.0 (3)	72.7 (7)	100 (9)	100 (8)	**<0.01**
Physical Abuse SLV	0.0 (0)	9.1 (1)	10.0 (1)	37.5 (3)	0.12
**1B. HIV-infected**					
**Variable**	**No Depression, No Abuse, %(N)**	**Depression Only, %(N)**	**Abuse Only, %(N)**	**Depression & Abuse, %(N)**	**p-value**
N	10	10	8	10	
**Demographics**					
Age, Median (IQR)	46.4 (43.8–54.1)	44.3 (39.3–46.0)	44.6 (40.9–48.2)	43.2 (36.4–52.0)	0.41
Race					0.84
White	10.0 (1)	20.0 (2)	12.5 (1)	10.0 (1)	.
African-American	80.0 (8)	60.0 (6)	75.0 (6)	90.0 (9)	.
Other	10.0 (1)	20.0 (2)	12.5 (1)	0.0 (0)	.
Education					**0.03**
<HS	0.0 (0)	70.0 (7)	37.5 (3)	50.0 (5)	.
HS	30.0 (3)	10.0 (1)	12.5 (1)	10.0 (1)	.
>HS	70.0 (7)	20.0 (2)	50.0 (4)	40.0 (4)	.
Income					0.34
<$12,000	33.3 (3)	50.0 (5)	57.1 (4)	80.0 (8)	.
<$24,000	22.2 (2)	40.0 (4)	28.6 (2)	10.0 (1)	.
>$24,000	44.4 (4)	10.0 (1)	14.3 (1)	10.0 (1)	.
Employed	70.0 (7)	30.0 (3)	25.0 (2)	0.0 (0)	**<0.01**
**Reproductive status and sexual behavior**					
# Male Sex Partners (last 6 months), Median (IQR)	1.0 (1.0–1.0)	1.0 (1.0–1.0)	1.0 (1.0–1.0)	1.50 (1.0–2.0)	**<0.01**
Vaginal Sex with Male last 48 hrs	20.0 (2)	0.0 (0)	12.5 (1)	0.0 (0)	.
Condom Use During Vaginal Sex					0.24
Always	70.0 (7)	57.1 (4)	50.0 (4)	22.2 (2)	.
Sometimes	0.0 (0)		25.0 (2)	33.3 (3)	.
Never	30.0 (3)	42.9 (3)	25.0 (2)	44.4 (4)	.
Anal Sex SLV	0.0 (0)	0.0 (0)	0.0 (0)	14.3 (1)	0.43
Transactional Sex SLV	0.0 (0)	0.0 (0)	0.0 (0)	10.0 (1)	1
BV by Amsel at visit	12.5 (1)	30.0 (3)	12.5 (1)	66.7 (6)	0.06
Been Through Menopause	20.0 (2)	0.0 (0)	12.5 (1)	20.0 (2)	0.57
Hormonal Contraception last 6 months	10.0 (1)	10.0 (1)	12.5 (1)	0.0 (0)	0.88
**Other risk behavior and abuse/depression status**					
Current Smoker	20.0 (2)	50.0 (5)	25.0 (2)	70.0 (7)	0.11
Ever use crack, cocaine, or heroin	10.0 (1)	30.0 (3)	62.5 (5)	90.0 (9)	**<0.01**
Drinks per week, Median (IQR)	0.0 (0.0–0.1)	0.0 (0.0–1.3)	0.4 (0.0–1.4)	1.5 (0.2–4.8)	0.07
CESD, Median (IQR)	0.0 (0.0–0.0)	36.5 (34.0–39.0)	4.5 (2.0–6.0)	38.5 (33.0–42.0)	**<0.01**
Any Abuse History	50.0 (5)	40.0 (4)	100 (8)	100 (10)	**<0.01**
Any Abuse SLV	0.0 (0)	0.0 (0)	12.5 (1)	40.0 (4)	**0.01**
Childhood Sexual Abuse	0.0 (0)	0.0 (0)	100 (8)	100 (10)	**<0.01**
Childhood Physical Abuse	20.0 (2)	10.0 (1)	37.5 (3)	66.7 (6)	0.06
IPV History	20.0 (2)	10.0 (1)	25.0 (2)	90.0 (9)	**<0.01**
IPV SLV	0.0 (0)	0.0 (0)	12.5 (1)	40.0 (4)	**0.01**
Sexual Abuse History	0.0 (0)	0.0 (0)	100 (8)	100 (10)	**<0.01**
Sexual Abuse SLV	0.0 (0)	0.0 (0)	0.0 (0)	30.0 (3)	**<0.01**
Physical Abuse History	50.0 (5)	40.0 (4)	87.5 (7)	100 (10)	**<0.01**
Physical Abuse SLV	0.0 (0)	0.0 (0)	0.0 (0)	30.0 (3)	**0.04**
**Clinical parameters**					
CD4 Count	475 (395–660)	739 (523–1086)	693 (557–782)	539 (445–702)	.
Log Viral Load	1.5 (1.3–2.6)	1.7 (1.7–1.8)	1.3 (1.3–2.7)	2.4 (1.3–4.0)	0.58
On HAART	100 (10)	100 (10)	100 (8)	100 (10)	.

Among HIV-infected participants (Table 1B), Control women were more likely to be educated (p = 0.03) and employed (p<0.01). Women in the Abuse+ Depression group had more male sexual partners in the past 6 months (p<0.01) and higher reported substance use (p<0.01). Women also differed significantly in terms of any abuse history (P<0.01), any abuse since last visit (p = 0.01), childhood sexual abuse, IPV history and IPV since last visit (p<0.01), physical abuse history and since last visit (p<0.01), in addition to the selection criteria variables of current depression and sexual abuse exposures (p<0.01). There were no differences in CD4 count, plasma viral load, and HAART usage between the groups.

For both HIV-uninfected and infected women, there were no differences by race, presence of bacterial vaginosis, BV status, or menopause (Table 1A and 1B).

### Changes in cervico-vaginal immune mediators by abuse or depression status in HIV-uninfected women

**Fig 1:**
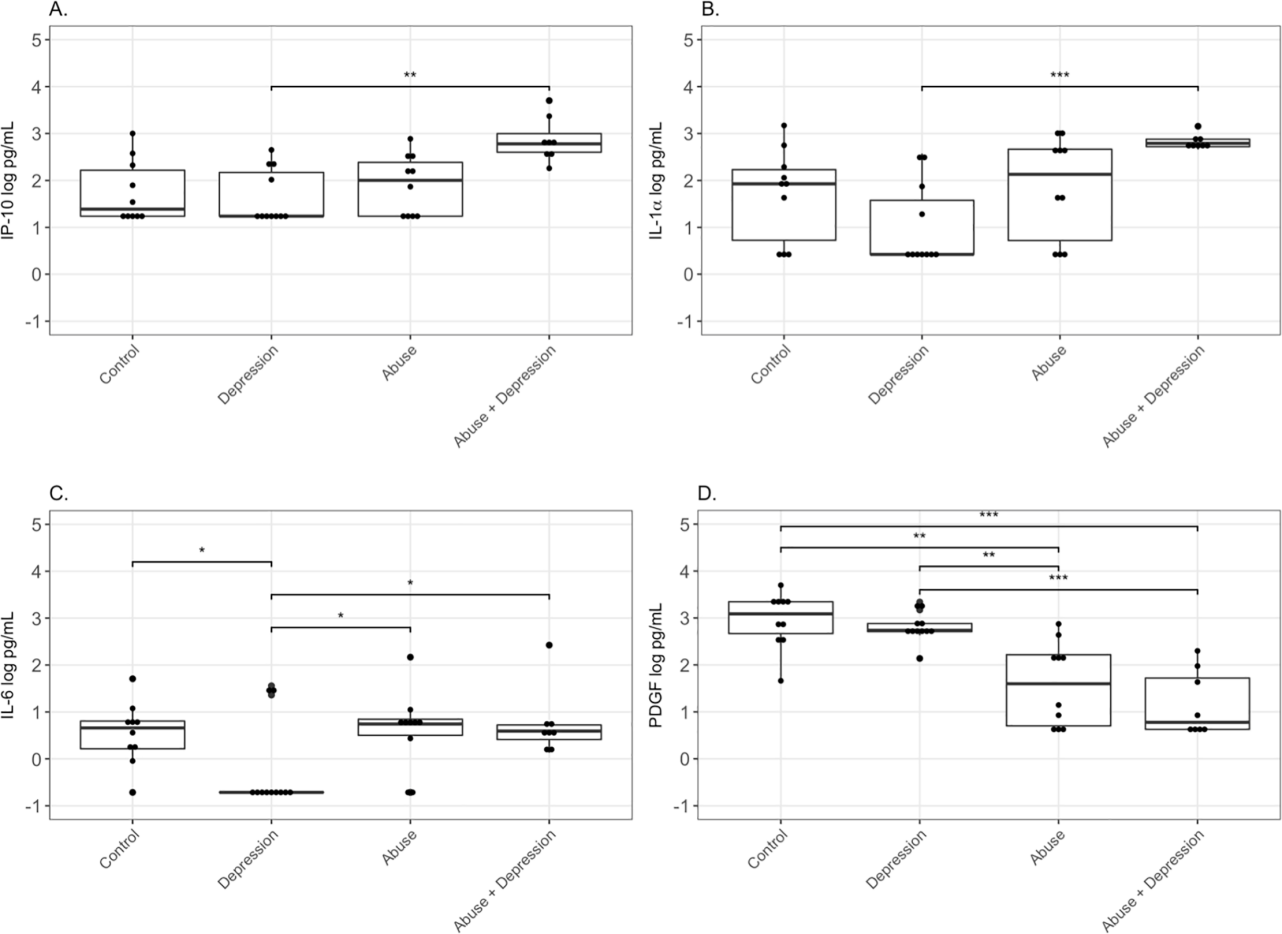
Differences in cervico-vaginal immune mediators by abuse status in HIV-uninfected women. CVL from Control women with no history of sexual abuse and depression (n = 10), women with current depression (n = 11), women with history of chronic sexual abuse (n = 10), and women with history of chronic sexual abuse and current depression (n = 8), were tested for levels of (A) IP-10, (B) IL-1α, (C) IL-6 and (D) PDGF by standard ELISA assays. Data is presented as log pg/mL of protein. Bars depict median. P value is indicated as follows: ***, p<0.0001; **, P<0.001; *,. P<0.05.

Median concentrations of the chemokine IP-10 were highest in the Abuse+Depression group (2.78 log pg/mL), followed by the Abuse (2.00 log pg/mL), and Control (1.39 log pg/mL) groups. The Depression group was mostly undetectable (1.24 log pg/mL) and significantly lower (p<0.01) (Fig 1A, Table 2A). A similar trend was observed for pro-inflammatory cytokine IL-1α levels with Abuse+Depression being the highest (2.79 log pg/mL), followed by the Abuse (2.13 log pg/mL) and the Control (1.93 log pg/mL) groups. The Depression group was mostly not detectable (0.42 log pg/mL) and significantly lower (p<0.01). (Fig 1B, Table 2A). Median values for another pro-inflammatory cytokine, IL-6, were also highest in the Abuse group and lowest in the Depression group (p = 0.04) (Fig 1C). In contrast, for the growth factor and wound healing mediator PDGF, highest median values were seen in the Control group (3.09 log pg/mL), followed by the Depression (2.74 log pg/mL) group and significantly lower levels in Abuse (1.60 log pg/mL) and Abuse+Depression (0.78 log pg/mL) groups (p<0.01) (Fig 1D, Table 2A).

**Table 2:** Levels of cervicovaginal immune mediators in HIV-uninfected (A) and HIV-infected (B) women with history of abuse, depression, or both abuse and depression. Data is presented in log pg/mL. Significant p-values denote differences in medians and are shown in bold. SLPI: secretory leukocyte protease inhibitor. HBD2: human beta defensin-2. HNP 1–3: human alpha defensin 1–3. TSP-1: Thrombospondin-1.

**2A. HIV Uninfected**						
**Variable (Log10 pg/mL)**	**Control Median (IQR)**	**Depression Median (IQR)**	**Abuse Median (IQR)**	**Abuse+Depression Median (IQR)**	**p value**	**p value After Protein Adjustment**
**n**	10	11	10	8		
**TNF-α**	-1.6 (-1.6–1.76)	0.89 (-1.6–2.24)	1.30 (-1.6–1.43)	1.25 (1.07–1.40)	0.53	0.23
**IL-6**	0.66 (0.18–0.81)	-0.71 (-0.71–0.71)	0.74 (0.44–0.86)	0.59 (0.37–0.74)	**0.04**	0.36
**IL-1α**	1.93 (0.42–2.29)	0.42 (0.42–1.87)	2.13 (0.42–2.67)	2.79 (2.72–2.88)	**<0.01**	**0.05**
**IL-1β**	1.58 (0.31–1.99)	0.31 (0.31–1.59)	1.15 (0.31–1.62)	1.76 (0.31–2.04)	0.19	0.35
**TGF-β**	0.26 (0.26–1.62)	1.56 (0.26–2.35)	0.26 (0.26–0.26)	0.26 (0.26–0.26)	**0.01**	**0.01**
**MIP-3α**	1.03 (-0.37–1.91)	0.90 (-0.67–1.36)	1.82 (1.05–1.88)	1.65 (1.10–1.89)	0.24	0.23
**IL-8**	2.62 (2.32–2.96)	2.51 (1.97–2.74)	2.63 (2.48–3.08)	2.82 (1.81–3.14)	0.82	0.87
**MCP-1**	1.27 (0.02–2.05)	1.77 (0.02–2.33)	0.02 (0.02–1.74)	0.02 (0.02–0.02)	0.13	0.06
**IP-10**	1.39 (1.24–2.32)	1.24 (1.24–2.32)	2.00 (1.24–2.43)	2.78 (2.56–3.12)	**<0.01**	**0.01**
**SLPI**	4.91 (4.54–5.15)	4.58 (4.00–5.04)	4.99 (4.31–5.34)	5.14 (4.35–5.51)	0.45	0.67
**Elafin**	5.34 (5.16–5.55)	5.25 (5.05–5.61)	5.15 (5.03–5.33)	5.36 (5.25–5.49)	0.32	0.86
**HBD-2**	3.73 (3.28–4.10)	3.64 (3.10–4.39)	3.38 (2.81–4.22)	3.73 (3.44–4.14)	0.71	0.78
**HNP1-3**	4.46 (3.70–5.15)	3.70 (3.70–4.95)	4.53 (4.38–5.46)	4.77 (3.96–5.09)	0.67	0.79
**TSP-1**	3.16 (2.83–3.32)	2.85 (2.62–3.00)	3.09 (2.87–3.32)	3.18 (3.03–3.53)	0.08	0.54
**Serpin A1**	5.50 (4.89–5.86)	4.68 (4.46–5.31)	5.20 (4.43–5.78)	5.54 (5.05–5.76)	0.2	0.08
**Serpin A5**	1.47 (1.47–1.47)	1.47 (1.47–2.61)	1.47 (1.47–2.40)	1.47 (1.47–2.23)	0.88	0.75
**Serpin B1**	2.87 (2.87–3.81)	2.87 (2.87–4.09)	4.01 (2.87–4.17)	4.02 (3.91–4.21)	0.14	0.15
**Cystatin A**	4.74 (4.58–5.47)	4.37 (3.53–5.75)	4.74 (4.37–5.06)	4.65 (3.23–5.58)	0.98	0.99
**Cystatin B**	4.40 (4.05–4.96)	2.86 (2.86–5.05)	2.86 (2.86–2.86)	4.40 (2.86–4.84)	0.09	0.08
**GRO-α**	1.97 (0.89–2.79)	1.19 (0.89–3.22)	2.60 (0.89–2.87)	2.69 (2.36–3.10)	0.5	0.35
**VEGF**	0.48 (0.48–0.48)	0.48 (0.48–0.48)	0.48 (0.48–2.02)	1.08 (0.48–2.08)	0.28	0.5
**PDGF**	3.09 (2.61–3.35)	2.74 (2.69–2.92)	1.60 (0.63–2.25)	0.78 (0.63–1.81)	**<0.01**	**<0.01**
**EGF**	1.30 (0.34–1.55)	0.56 (-.24–0.88)	-0.24 (-0.24–1.47)	-0.24 (-0.24–0.49)	0.12	0.19
**FGF**	1.28 (1.28–1.28)	1.28 (1.28–1.28)	1.28 (1.28–1.28)	1.28 (1.28–1.92)	**<0.01**	0.61
**IGF**	1.29 (1.29–2.36)	1.29 (1.29–1.29)	1.29 (1.29–2.05)	1.94 (1.29–2.87)	0.36	0.63
**Cathepsin B**	4.21 (4.03–4.52)	4.19 (3.77–4.41)	4.13 (3.80–4.30)	4.05 (3.84–4.24)	0.67	0.38
**Protein (Log10 ug/mL)**	2.12 (1.84–2.45)	1.99 (1.65–2.38)	2.13 (1.60–2.43)	2.13 (1.80–2.45)	0.85	
**% HIV inhibition (BaL)**	33.54 (25.52–43.12)	38.97 (30.74–52.20)	48.86 (45.93–61.20)	59.30 (44.76–62.42)	0.26	
**% HIV inhibition (RHPAc)**	28.30 (17.08–48.74)	38.40 (20.41–43.91)	39.25 (34.72–49.39)	34.17 (31.73–37.29)	0.53	
**2B. HIV Infected**						
**Variable (Log10 pg/mL)**	**Control Median (IQR)**	**Depression Median (IQR)**	**Abuse Median (IQR)**	**Abuse+Depression Median (IQR)**	**p value**	**p value After Protein Adjustment**
**n**	10	10	8	10		
**TNF-α**	1.12 (-1.3–1.40)	1.47 (1.18–1.52)	0.43 (-1.6–1.00)	-1.6 (-1.6–0.06)	**<0.01**	**<0.01**
**IL-6**	0.58 (0.27–1.00)	0.97 (0.86–1.16)	1.08 (1.01–1.24)	1.10 (0.88–1.14)	**0.05**	0.33
**IL-1α**	2.73 (2.46–2.83)	2.15 (1.69–2.81)	0.88 (0.58–1.71)	1.94 (0.42–2.23)	0.06	**0.01**
**IL-1β**	0.92 (0.31–1.89)	0.86 (0.31–1.06)	1.15 (0.31–1.63)	1.31 (0.31–1.88)	0.82	0.19
**TGF-β**	0.26 (0.26–0.26)	0.66 (0.26–1.71)	0.26 (0.26–1.73)	0.41 (0.26–1.57)	0.12	0.27
**MIP-3α**	1.36 (1.14–1.66)	1.05 (-0.67–1.27)	-0.67 (-0.67–1.76)	-0.67 (-0.67–0.67)	**<0.01**	**<0.01**
**IL-8**	2.36 (2.15–2.93)	2.33 (1.95–2.93)	2.49 (2.27–3.26)	2.39 (1.67–2.87)	0.79	0.32
**MCP-1**	0.02 (0.02–1.94)	2.36 (1.68–2.58)	2.58 (2.28–2.75)	2.21 (1.78–2.54)	**0.01**	0.15
**IP-10**	2.90 (2.41–3.19)	2.72 (2.56–3.06)	2.36 (2.12–2.72)	2.20 (2.04–2.92)	0.51	0.27
**SLPI**	4.72 (4.33–5.14)	4.96 (4.54–5.38)	5.17 (4.66–5.54)	4.44 (4.08–4.83)	0.28	0.56
**Elafin**	5.46 (5.10–5.57)	5.47 (5.08–5.72)	5.28 (5.04–5.66)	5.28 (5.06–5.40)	0.56	0.64
**HBD-2**	3.48 (3.04–4.43)	3.62 (3.17–4.15)	3.63 (2.96–4.21)	3.03 (2.69–4.34)	0.45	0.58
**HNP1-3**	4.10 (3.70–5.11)	3.70 (3.70–4.00)	3.90 (3.70–4.70)	3.70 (3.70–4.49)	0.64	0.25
**TSP-1**	3.06 (2.93–3.41)	3.03 (2.98–3.36)	3.01 (2.94–3.52)	3.02 (2.89–3.11)	0.72	0.55
**Serpin A1**	5.05 (4.89–5.53)	5.54 (5.19–5.80)	5.53 (5.20–5.67)	4.99 (4.43–5.97)	0.27	0.19
**Serpin A5**	1.47 (1.47–2.71)	1.47 (1.47–3.05)	2.70 (1.78–3.48)	2.89 (1.77–3.10)	0.15	0.29
**Serpin B1**	2.87 (2.87–4.19)	3.02 (2.87–3.63)	3.59 (2.87–4.42)	4.07 (3.31–4.26)	0.20	0.23
**Cystatin A**	4.54 (3.23–5.55)	3.23 (3.23–4.15)	3.23 (3.23–4.73)	3.23 (3.23–4.62)	0.36	0.1
**Cystatin B**	4.37 (3.58–4.53)	3.27 (2.86–4.17)	4.52 (3.64–4.92)	4.24 (3.51–4.78)	0.24	0.15
**GRO-α**	2.39 (0.89–2.82)	2.47 (0.89–2.55)	2.86 (2.61–3.31)	2.79 (2.62–3.07)	0.06	**0.03**
**VEGF**	0.79 (0.48–2.13)	1.76 (0.48–2.35)	2.06 (1.65–2.34)	1.94 (1.62–2.28)	0.61	0.35
**PDGF**	0.96 (0.63–1.75)	1.57 (0.63–2.78)	2.13 (1.79–2.22)	1.55 (0.63–2.41)	0.42	0.76
**EGF**	-.24 (-.24–1.73)	0.87 (-.24–1.00)	0.42 (-.24–1.20)	0.56 (-.24–1.21)	0.97	0.75
**FGF**	1.73 (1.28–1.95)	2.39 (1.28–2.70)	2.20 (1.28–2.61)	1.28 (1.28–1.58)	0.19	0.78
**IGF**	1.65 (1.29–2.63)	1.29 (1.29–1.29)	1.29 (1.29–2.32)	1.29 (1.29–1.29)	0.32	0.54
**Cathepsin B**	4.13 (3.78–4.32)	4.06 (3.77–4.20)	3.56 (3.10–4.10)	4.07 (3.51–4.19)	0.37	**<0.01**
**Protein (Log10 ug/mL)**	2.13 (1.75–2.34)	2.25 (2.09–2.34)	2.41 (2.16–2.65)	2.05 (1.66–2.39)	0.24	
**% HIV inhibition (BaL)**	59.92 (46.58–75.28)	74.05 (68.12–94.12)	46.33 (39.70–78.35)	63.92 (46.12–80.20)	0.24	
**% HIV inhibition (RHPAc)**	44.53 (31.67–67.46)	67.06 (51.75–95.42)	49.14 (29.21–66.02)	67.31 (61.80–81.73)	0.15	
**CD4 Count**	475 (395–660)	739 (523–1086)	693 (557–782)	539 (445–702)	0.06	
**Log Viral Load**	1.46 (1.30–2.57)	1.69 (1.67–1.75)	1.30 (1.30–2.62)	2.42 (1.30–4.00)	0.58	

Median concentrations of the anti-inflammatory cytokine TGF-β were highest in the Depression group (1.78 log pg/mL), followed by significantly lower and mostly undetectable values for Control (0.26 log pg/mL), Abuse (0.26 log pg/mL) and Abuse+Depression groups (0.26 log pg/mL) (p<0.01) (Table 2A).

Using linear regression analysis, current depressive symptoms (after controlling for chronic sexual abuse), was not significantly associated with any difference in levels of biomarkers (Table 3A). However, chronic sexual abuse (after controlling for depressive symptoms) was associated with a 0.68 pg/mL log increase of MIP-3α (p = 0.04), 0.95 pg/mL log increase of IL-1α (p = 0.01), 0.67 pg/mL log increase of IP-10 (p<0.01), and a 0.53 pg/mL log increase of Serpin B1 (p = 0.01) (Table 3A). Chronic sexual abuse was also associated with a 0.76 pg/mL log decrease of MCP-1 (p = 0.02), 1.49 pg/mL log decrease of PDGF (p<0.01), and a 0.71 pg/mL log decrease in TGF-β (p<0.01) (Table 3A). Since CVL samples can have variable protein content [43,44], we measured total protein in each CVL sample and adjusted each mediator to the protein content in that. All values remained significant before and after protein adjustment except IL-6 and FGF (Tables 2A and 3A). Interaction between chronic sexual abuse and current depressive symptoms was significant for IL-1α, IP-10, Cystatin B, Serpin A1 and FGF (before protein adjustment), which suggests that the relationship between chronic sexual abuse and these markers differs by depressive symptoms, or vice versa (Table 3A). FGF did not remain significant following protein adjustment (Table 3A).

**Table 3:** Main effects and Interaction models for the effects of depression and abuse on FRT biomarkers in HIV-uninfected (A) and HIV-infected (B) women. Linear regression models were used. Abuse defined as childhood sexual abuse and adult sexual abuse reported at baseline.

**3A. HIV Uninfected**											
	**Model without Interaction**						**Model with Interaction**				
**Outcome (Log10 pg/mL)**	**Depressive Symptoms vs None**	**p value**	**p value after Protein Adjustment**	**Abuse vs None**	**p value**	**p value after Protein Adjustment**	**Depressive Symptoms vs None**	**Abuse vs None**	**Depression-Abuse Interaction**	**p value**	**p value after Protein Adjustment**
**TNF-α**	0.74	0.15	0.14	0.94	0.07	0.10	0.89	1.09	-0.32	0.75	0.67
**IL-6**	-0.40	0.13	0.19	0.54	0.05	0.09	-0.86	0.06	1.00	0.06	0.11
**IL-1α**	0.07	0.82	0.67	0.95	**<0.01**	**<0.01**	-0.69	0.15	1.67	**0.01**	**<0.01**
**IL-1β**	-0.25	0.37	0.44	0.12	0.66	0.84	-0.71	-0.36	1.01	0.06	0.10
**TGF-β**	0.18	0.46	0.42	-0.71	**<0.01**	**0.01**	0.55	-0.33	-0.80	0.09	0.08
**MIP-3α**	-0.02	0.96	0.94	0.68	**0.04**	**0.05**	-0.22	0.47	0.44	0.50	0.61
**IL-8**	-0.24	0.41	0.47	-0.12	0.69	0.51	-0.44	-0.32	0.42	0.47	0.59
**MCP-1**	0.02	0.94	0.84	-0.76	**0.02**	**0.01**	0.32	-0.45	-0.64	0.33	0.23
**IP-10**	0.37	0.08	0.08	0.67	**<0.01**	**0.01**	-0.11	0.16	1.06	**0.01**	**0.04**
**SLPI**	-0.15	0.54	0.63	0.27	0.25	0.35	-0.33	0.08	0.40	0.41	0.55
**Elafin**	0.02	0.84	0.66	-0.02	0.84	0.54	-0.14	-0.19	0.35	0.13	0.45
**HBD-2**	0.04	0.88	0.70	-0.04	0.86	0.60	-0.20	-0.29	0.51	0.27	0.35
**HNP1-3**	-0.20	0.40	0.45	0.23	0.35	0.45	-0.28	0.15	0.17	0.73	0.92
**TSP-1**	0.06	0.69	0.52	0.25	0.09	0.24	-0.08	0.10	0.30	0.30	0.57
**Serpin A1**	-0.12	0.61	0.62	-0.01	0.97	0.64	-0.54	-0.45	0.91	**0.05**	**0.01**
**Serpin A5**	0.13	0.54	0.53	0.04	0.84	0.94	0.23	0.14	-0.21	0.64	0.55
**Serpin B1**	0.14	0.46	0.34	0.53	**0.01**	**0.02**	0.08	0.46	0.13	0.73	0.99
**Cystatin A**	-0.04	0.89	0.99	-0.10	0.76	0.65	-0.08	-0.14	0.08	0.90	0.95
**Cystatin B**	-0.09	0.80	0.88	-0.29	0.39	0.26	-0.87	-1.12	1.71	**0.01**	**0.01**
**GRO-α**	0.17	0.61	0.49	0.54	0.11	0.13	-0.16	0.20	0.72	0.29	0.34
**VEGF**	-0.08	0.76	0.84	0.37	0.16	0.14	-0.24	0.21	0.34	0.53	0.62
**PDGF**	-0.28	0.18	0.25	-1.49	**<0.01**	**<0.01**	-0.16	-1.36	-0.26	0.53	0.35
**EGF**	-0.47	0.07	0.14	-0.39	0.14	0.13	-0.53	-0.44	0.12	0.82	0.99
**FGF**	0.13	0.05	0.26	0.14	**0.04**	0.64	0.00	0.00	0.28	**0.03**	0.61
**IGF**	0.14	0.55	0.49	0.23	0.33	0.54	-0.09	-0.01	0.49	0.30	0.49
**Cathepsin B**	-0.10	0.51	0.66	-0.15	0.34	0.11	-0.16	-0.21	0.12	0.69	0.98
**3B. HIV Infected**											
	**Model without Interaction**						**Model with Interaction**				
**Outcome (Log10 pg/mL)**	**Depressive Symptoms vs None**	**p value**	**p value after Protein Adjustment**	**Abuse vs None**	**P value**	**p value after Protein Adjustment**	**Depressive Symptoms vs None**	**Abuse vs None**	**Depression-Abuse Interaction**	**p value**	**p value after Protein Adjustment**
**TNF-α**	-0.06	0.89	0.96	-1.27	**<0.01**	**<0.01**	0.57	-0.56	-1.33	0.11	0.36
**IL-6**	0.04	0.79	0.49	0.36	0.08	**0.03**	0.26	0.61	-0.47	0.16	0.69
**IL-1α**	0.09	0.77	0.58	-0.71	**0.02**	**0.02**	-0.26	-1.11	0.74	0.22	**0.02**
**IL-1β**	-0.10	0.69	0.92	0.25	0.41	0.31	-0.21	0.12	0.24	0.63	0.07
**TGF-β**	0.32	0.19	0.11	0.16	0.64	0.50	0.72	0.62	-0.86	0.07	0.59
**MIP-3α**	-1.00	**<0.01**	**<0.01**	-1.16	**<0.01**	**<0.01**	-1.00	-1.15	0.00	0.99	0.31
**IL-8**	-0.38	0.17	0.18	0.08	0.91	0.78	-0.33	0.13	-0.11	0.85	0.28
**MCP-1**	0.34	0.29	0.21	0.79	**0.03**	**0.02**	1.01	1.54	-1.42	**0.02**	0.21
**IP-10**	-0.04	0.86	0.88	-0.21	0.32	0.30	0.00	-0.17	-0.08	0.85	0.33
**SLPI**	-0.05	0.83	0.89	-0.05	0.64	0.84	0.26	0.30	-0.65	0.16	0.92
**Elafin**	-0.06	0.75	0.91	-0.13	0.31	0.47	0.10	0.05	-0.33	0.35	0.43
**HBD-2**	-0.05	0.84	0.92	-0.30	0.13	0.25	0.16	-0.06	-0.44	0.40	0.72
**HNP1-3**	-0.30	0.22	0.32	0.01	0.87	0.96	-0.37	-0.07	0.16	0.75	0.09
**TSP-1**	-0.07	0.55	0.96	-0.06	0.49	0.62	0.04	0.06	-0.22	0.33	0.21
**Serpin A1**	0.04	0.84	0.48	-0.19	0.16	0.38	0.40	0.22	-0.76	0.07	0.64
**Serpin A5**	0.06	0.81	0.64	0.53	0.11	0.05	0.16	0.64	-0.20	0.71	0.50
**Serpin B1**	0.04	0.85	0.64	0.44	0.11	**0.04**	-0.04	0.36	0.16	0.70	0.11
**Cystatin A**	-0.43	0.19	0.23	-0.26	0.29	0.43	-0.79	-0.67	0.77	0.23	**0.01**
**Cystatin B**	-0.37	0.14	0.30	0.39	0.25	0.13	-0.54	0.20	0.36	0.49	0.09
**GRO-α**	-0.06	0.81	0.96	0.75	**0.01**	**0.01**	0.00	0.82	-0.13	0.81	0.33
**VEGF**	0.07	0.80	0.57	0.41	0.17	0.17	0.29	0.66	-0.46	0.44	0.78
**PDGF**	-0.09	0.76	0.95	0.32	0.40	0.30	0.28	0.74	-0.79	0.21	0.78
**EGF**	0.09	0.74	0.53	-0.05	0.71	0.86	0.15	0.02	-0.13	0.81	0.38
**FGF**	0.07	0.76	0.54	-0.07	0.60	0.75	0.48	0.40	-0.88	**0.04**	0.56
**IGF**	-0.37	0.07	0.28	-0.08	0.64	0.70	-0.50	-0.22	0.26	0.51	0.11
**Cathepsin B**	0.14	0.40	0.16	-0.30	**0.03**	0.08	-0.01	-0.47	0.33	0.33	**<0.01**

### Changes in cervico-vaginal immune mediators by abuse or depression status in HIV-infected women

**Fig 2:**
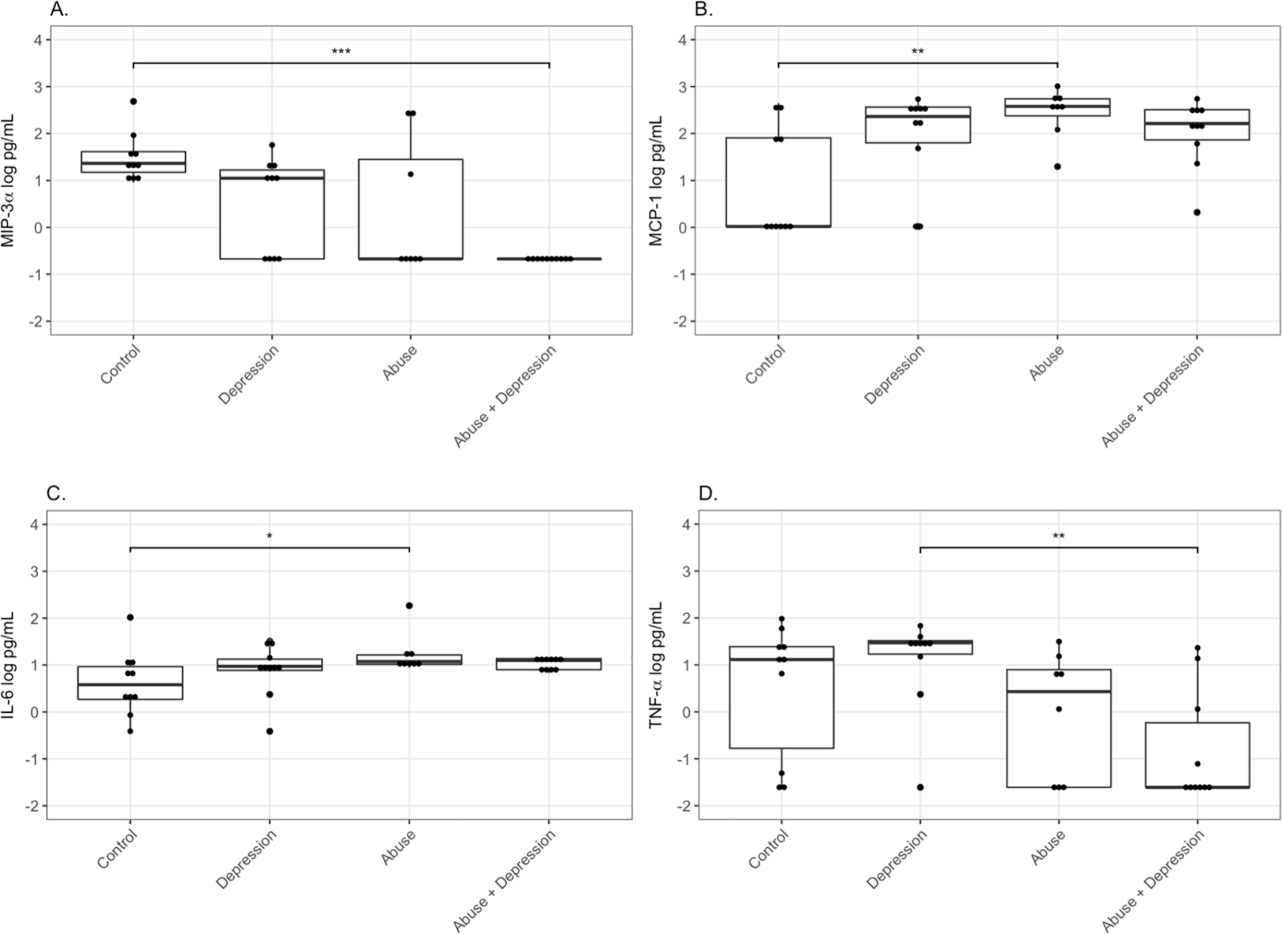
Differences in cervico-vaginal immune mediators by abuse status in HIV-infected women. CVL from Control women with no history of sexual abuse and depression (n = 10), women with current depression (n = 10), women with history of chronic sexual abuse (n = 8), and women with history of chronic sexual abuse and current depression (n = 10), were tested for levels of (A) MIP-3α, (B) MCP-1, (C) IL-6, (D) TNF-α, by standard ELISA assays. Data is presented as log pg/mL of protein. Bars depict median. P value is indicated as follows: ***, p<0.0001, **, P<0.001, *, P<0.05.

Median concentrations of the chemokine MIP-3α were highest in the Control group (1.36 log pg/mL), followed by Depression (1.05 log pg/mL) and mostly undetectable levels in Abuse (-0.67 log pg/mL) and Abuse+Depression groups (-0.67 log pg/mL) (p<0.01) (Fig 2A, Table 2B). For another chemokine MCP-1, median concentrations were highest in the Abuse group (2.58 log pg/mL), followed by Depression (2.36 log pg/mL) and Abuse+Depression (2.21 log pg/mL) with mostly undetectable values for Controls (p = 0.01) (Fig 2B, Table 2B). Concentrations of the pro-inflammatory cytokine IL-6 were highest in the Abuse+Depression group (1.10 log pg/mL), followed by Abuse (1.08 log pg/mL), Depression (0.97 log pg/mL) and Control (0.58 log pg/mL) (p = 0.05) (Fig 2C, Table 2B). Median concentrations of another pro-inflammatory cytokine TNF-α were highest in the Depression (1.47 log pg/mL) group, followed by the Control (1.12 log pg/mL) and Abuse (0.43 log pg/mL) groups with mostly undetectable values for the Abuse+Depression group (p<0.01) (Fig 2D, Table 2B).

Differences observed for MIP-3α and TNF-α remained significant following protein adjustment (Table 2B). However, IL-6 and MCP-1 were no longer significant (Table 2B). On the other hand, Cathepsin B, IL-1α, and Gro-α were not significantly different before protein adjustment, but did show significant differences after adjustment (Table 2B). Cathepsin B and IL-1α concentrations were the lowest in the Abuse group (p<0.01, and p = 0.01, respectively), whereas Gro-α concentrations were highest in the Abuse group (p = 0.03) (Table 2B).

Using linear regression analysis, following adjustment for CD4 and viral load, depressive symptoms (after controlling for chronic sexual abuse) were associated with 1.00 pg/mL log decrease of MIP-3α (p<0.01). Chronic sexual abuse (after controlling for depressive symptoms), was associated with a 0.79 pg/mL log increase of MCP-1 (p = 0.03), and a 0.75 pg/mL log increase in Gro-α (p = 0.01). Chronic sexual abuse was also associated with a 1.16 pg/mL log decrease of MIP-3α (p<0.01), 1.27 pg/mL log decrease of TNF-α (p<0.01), 0.71 pg/mL log decrease of IL-1α (p = 0.02) and 0.30 pg/mL log decrease in Cathepsin B (p = 0.03) (Table 3B). Adjustment to total protein did not change these results except increased IL-6 (p = 0.03) and Serpin B1 (p = 0.04) were now significantly associated with chronic sexual abuse. Interaction between chronic sexual abuse and depressive symptoms was significant for MCP-1 and FGF before, but not after protein adjustment (Table 3B). Following protein adjustment, significant interactions were noted for Cathepsin B (p<0.01), IL-1α (p = 0.02) and Cystatin A (p = 0.01) (Table 3B).

### Effect modifications by HIV status

In analyzing the differences between HIV uninfected and HIV infected women by depression or abuse status (Table 4), we observed the association between MIP-3α and depression was significantly lower (p = 0.03) in HIV infected women compared to HIV uninfected women. The association between MIP-3α and abuse was also significantly lower (p<0.01) in HIV infected compared to HIV uninfected women. Whereas none of the other mediators showed a differential association with depression by HIV status, the association between abuse and TNF-α (p<0.01), IL-1α (p<0.01) and IP-10 (p<0.01) was significantly lower in HIV infected compared to uninfected women. Conversely, the association between abuse and MCP-1 (p<0.01) and PDGF (p<0.01) was significantly higher in HIV infected compared to uninfected women.

**Table 4:** Main effects and interaction models for the effects of depression and abuse on systemic biomarkers, by HIV status. Linear regression models were used. Abuse defined as childhood sexual abuse and adult sexual abuse reported at baseline.

Outcome (Log10 pg/mL)	Change in Outcome, Depression vs None	Change in Outcome, Abuse vs None	Change in Outcome, HIV+ vs HIV	HIV Abuse Interaction	p value	HIV Depression Interaction	p value
**TNF-α**	0.74	0.94	1.26	-2.21	**<0.01**	-0.79	0.23
**IL-6**	-0.40	0.54	0.43	-0.18	0.57	0.45	0.15
**IL-1α**	0.07	0.95	0.82	-1.66	**<0.01**	0.02	0.97
**IL-1β**	-0.25	0.12	-0.21	0.13	0.73	0.15	0.68
**TGF-β**	0.18	-0.71	-0.58	0.88	**0.01**	0.14	0.68
**MIP-3α**	-0.02	0.68	0.73	-1.84	**<0.01**	-0.98	**0.03**
**IL-8**	-0.24	-0.12	0.03	0.19	0.63	-0.14	0.72
**MCP-1**	0.02	-0.76	-0.04	1.55	**<0.01**	0.31	0.49
**IP-10**	0.37	0.67	1.12	-0.88	**<0.01**	-0.41	0.16
**SLPI**	-0.15	0.27	0.06	-0.32	0.33	0.10	0.76
**Elafin**	0.02	-0.02	0.10	-0.10	0.62	-0.08	0.70
**HBD-2**	0.04	-0.04	0.11	-0.26	0.46	-0.09	0.80
**HNP1-3**	-0.20	0.23	-0.18	-0.21	0.53	-0.10	0.77
**TSP-1**	0.06	0.25	0.30	-0.30	0.10	-0.13	0.49
**Serpin A1**	-0.12	-0.01	0.16	-0.18	0.57	0.16	0.61
**Serpin A5**	0.13	0.04	0.27	0.49	0.15	-0.07	0.84
**Serpin B1**	0.14	0.53	0.02	-0.09	0.76	-0.10	0.72
**Cystatin A**	-0.04	-0.10	-0.35	-0.16	0.72	-0.38	0.40
**Cystatin B**	-0.09	-0.29	0.08	0.68	0.11	-0.29	0.49
**GRO-α**	0.17	0.54	0.36	0.21	0.62	-0.23	0.59
**VEGF**	-0.08	0.37	0.53	0.04	0.92	0.16	0.69
**PDGF**	-0.28	-1.49	-1.46	1.81	**<0.01**	0.18	0.62
**EGF**	-0.47	-0.39	-0.41	0.34	0.36	0.57	0.13
**FGF**	0.13	0.14	0.65	-0.21	0.37	-0.06	0.78
**IGF**	0.14	0.23	0.31	-0.31	0.32	-0.51	0.10
**Cathepsin B**	-0.10	-0.15	-0.22	-0.15	0.51	0.24	0.29

### Anti-HIV activity

Previously, we have demonstrated intrinsic HIV inhibitory activity in CVL and its association with immune mediators as well as clinical parameters such as CD4 counts [45,46]. Using the TZM-bl assay for HIV infection, we investigated whether abuse or depression status impacts functional anti-HIV activity in CVL. Although we observed a wide range of inhibition in all eight groups, we did not find significant differences in anti-HIV activity based on abuse or depression status in either HIV-infected or HIV-uninfected women (Table 1A and 1B). This observation held true for both inhibitory activity against the laboratory-adapted strain BaL, as well as the heterosexually transmitted T/F strain RHPA.c (Table 1A and 1B). As expected, CVL from the HIV-infected individuals in our cohort showed better inhibitory activity compared to the HIV-uninfected individuals, as they were all on HAART. Interestingly, however, several HIV-infected individuals did not inhibit the virus at all in spite of being on HAART. This could point toward lack of adherence or general dysregulation of FRT immunity.

### Changes in immune mediator (cytokine/chemokine/antimicrobial) network

**Fig 3:**
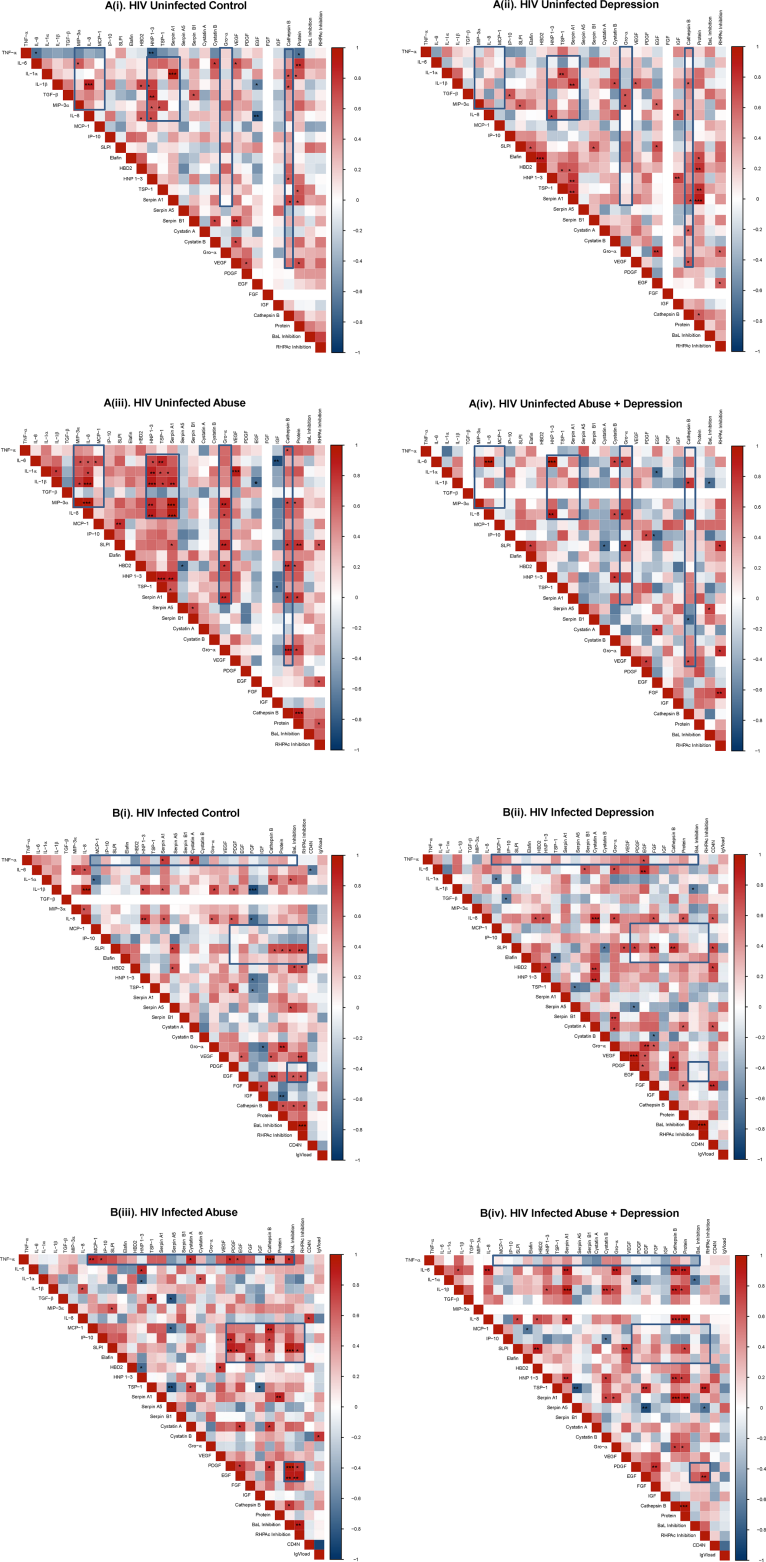
**Heat map of Spearman’s coefficients for HIV-uninfected (A) and HIV-infected (B) samples stratified by abuse and depression status.** Distinct associative patterns were observed among (A i-iv) HIV negative Control, Depression, Abuse, Abuse+Depression as well as (B i-iv) HIV-infected Controls, Depression, Abuse and Abuse+Depression. Each cell of the heat map denotes the Spearman correlation coefficients. Cells highlighted in shades of RED indicates a positive association with darker shades representing stronger associations. Similarly, cells highlighted in shades of BLUE indicates a negative association with darker shades representing stronger associations. Statistically significant (p<0.05) associations are denoted in BOLD. Missing numbers (white) indicate there was no variation within that subgroup usually due to undetectable values, therefore the correlation coefficient could not be calculated. Boxed-in sections highlight clustering patterns.

Distinct immune clusters have been described previously in the context of HIV infection and susceptibility [47]; associations between immune mediators are important to consider as they can interact synergistically or antagonistically and be clinically predictive [48–51]. For soluble immune mediators tested, we saw significantly altered clustering patterns in Abuse groups compared to Control in both HIV-uninfected (Fig 3A i-iv) and HIV-infected populations (Fig 3B i-iv).

In HIV-uninfected Abuse (Fig 3A, iii), strong positive interactions were observed between chemokines (MIP-3α, IL-8, MCP-1) and cytokines (IL-6, IL-1α, IL-1β). A strong grouping of positive interactions was also observed between these cytokines and chemokines with anti-HIV factors HNP 1–3, TSP-1 and Serpin A1. Finally, Gro-α and Cathepsin B associated significantly with multiple immune biomarkers. None of these patterns (shown as boxes on the heat-maps) were present in Control, Depression and Abuse+Depression groups (Fig 3A i, ii, iv). Overall, the Abuse group showed the maximum number of significant associations (47 positive and 4 negative).

Associations among biomarkers were distinct in the HIV-infected population (Fig 3B i-iv). In the Abuse group, strong positive interactions were observed between the pro-inflammatory cytokine TNF-α and chemokines (MCP-1, IP-10), anti-HIV mediators (Cystatin A), growth factors (PDGF, EGF), protease (Cathepsin B) and HIV BaL inhibition. There was also a strong cluster between growth factors (PDGF, EGF, FGF, IGF) with chemokines (MCP-1, IP-10), anti-HIV factors (SLPI, Elafin) and protease (Cathepsin B). PDGF and EGF also associated significantly with inhibition of HIB BaL and RHPA. As with the HIV uninfected, none of the associations in Abuse (shown as boxes on the heat-maps) were present in Control, Depression, and Abuse+Depression (Fig 3B, i, ii, iv). Interestingly, in both HIV-uninfected and infected categories, immune associations in Abuse+Depression did not resemble the patterns in either Abuse or Depression.

## Discussion

**Fig 4:**
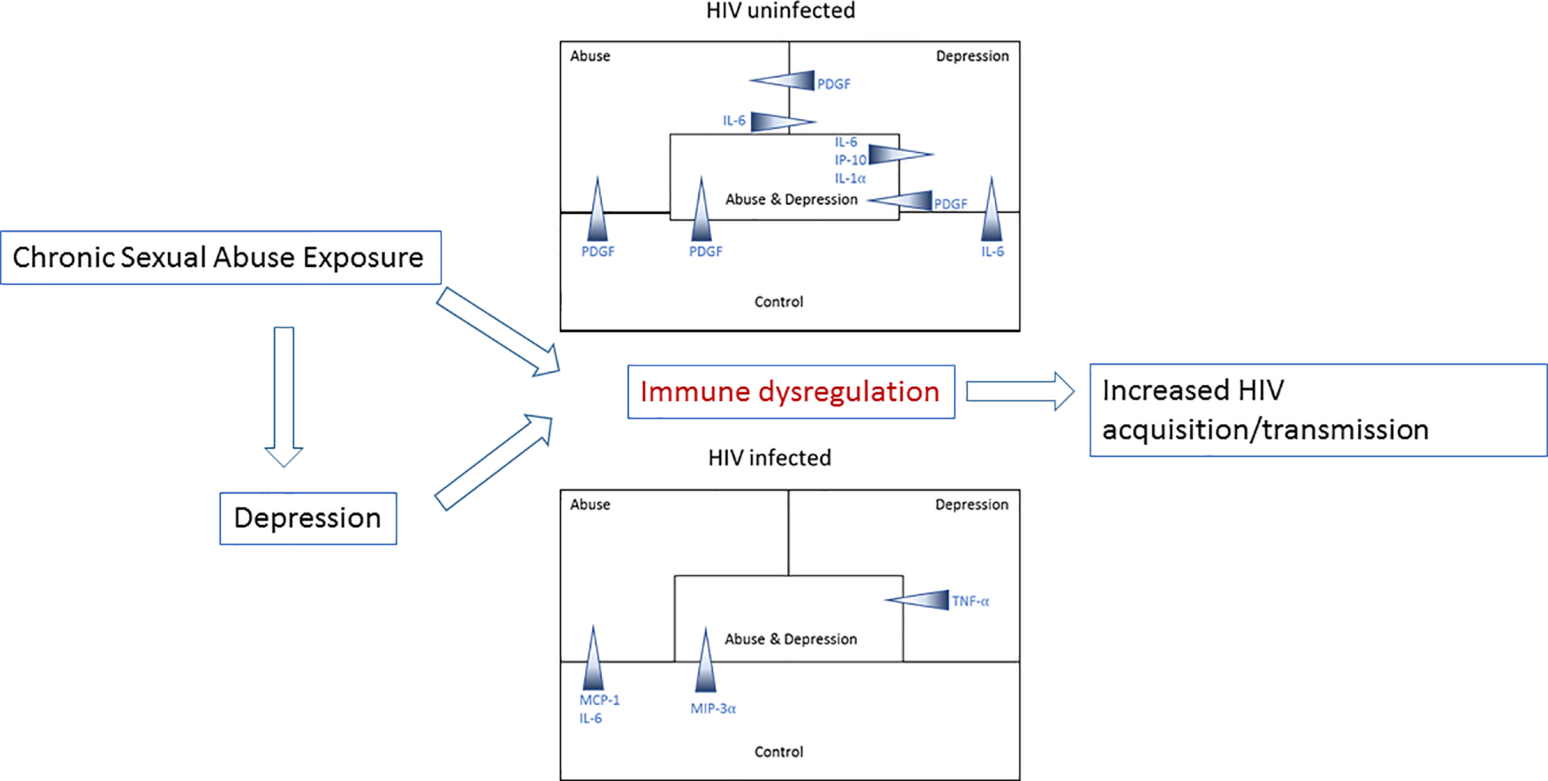
Hypothetical model of interaction between exposure to chronic sexual abuse, depression, immune dysregulation and HIV susceptibility. Our model hypothesizes that exposure to sexual abuse can directly result in immune dysregulation leading to increased HIV susceptibility. Depression resulting from exposure to sexual abuse can also lead to immune dysregulation and increased HIV susceptibility. Our major findings are also summarized schematically. Immune mediators are shown as gradients with the wider end of the arrows denoting groups where the concentrations were higher.

Our data indicate that exposure to chronic sexual abuse and current depressive symptoms can be associated with immune alterations which can include a state of inflammation, immune activation, and modified wound healing pathways within the FRT, thereby increasing risk of HIV acquisition/transmission. These findings have clinical implications in HIV prevention efforts, as therapies that abate inflammation and immune activation (such as using anti-inflammatory drugs, [52]) and modulate wound healing [53], could be applied to reduce risks of HIV infection in women who have histories of abuse and depression. Fig 4 shows a schematic of our hypothesis and major findings.

In HIV-uninfected women, we found significantly higher levels of pro-inflammatory cytokine IL-1α and chemokine IP-10 in Abuse+Depression group compared to Controls, indicating inflammation/immune activation. This was coupled with significantly lower levels of PDGF in both Abuse and Abuse+Depression groups compared to Controls. Growth factors, such as PDGF, play a critical role in early stages of wound healing by initiating the process of formation of blood clots [14], [12]. Genital wound healing has been associated with hormonal contraceptive usage, as well as menopause [8]. Impaired wound healing in the HIV-infected population has been described in cases of adult male circumcision [54] but little is known regarding biomarkers of wound healing in women experiencing chronic sexual abuse. Our finding of decreased PDGF in women experiencing chronic sexual abuse suggests wound healing mechanisms might not be optimally functional in this population. Our linear regression models also confirmed associations between chronic sexual abuse and increases in inflammation/immune activation biomarkers (MIP-3α, IP-10, and IL-1α) along with decreases in factors associated with wound-healing (PDGF and TGF-β). TGF-β is a pleiotropic cytokine that is involved in all stages of wound repair [55]. As HIV is known to show enhanced infection in presence of genital inflammation and epithelial breach [13], [56] increased inflammation (higher IL-1α and IP-10), coupled with impaired wound healing could be detrimental for these women in terms of HIV acquisition.

In HIV-infected women, we found significantly increased levels of IL-6 and MCP-1 in Abuse groups compared to Controls, which can be indicative of inflammation and immune activation. Increased levels of IL-6 in HIV-infected women has been shown to be associated with inflammation and HIV genital tract shedding, resulting in increased risk for HIV transmission [57], [58]. MCP-1 has been associated with chronic mucosal inflammation and increased levels have been reported in HIV infected individuals [59], [60], [61], [62]. In contrast, we observed significantly decreased levels of pro-inflammatory cytokines IL-1α, TNF-α and chemokine MIP-3α in Abuse+Depression groups compared to Controls. It is possible that since all the HIV-infected women were on HAART, certain inflammatory pathways were suppressed. However, it has also been shown that HAART does not completely suppress systemic chronic inflammation [21]. Our data confirms and extends these findings in the FRT showing suppression of certain inflammatory pathways, but not others in HIV-infected women experiencing chronic sexual abuse. Further, MIP-3α also functions as an anti-HIV antimicrobial [63] and therefore its significant downregulation in Abuse+Depression groups can imply lack of protection against HIV.

While most studies quantify levels of individual immune mediators, changes observed at the individual level do not reflect how these mediators interact with each other *in vivo* where they are present concurrently, in a given tissue or biological fluid. Previous reports have shown that immune mediators can interact synergistically or antagonistically and be clinically predictive [48–51]. A heat-map analysis is designed to show correlations or associations between immune mediators which implies cross-talk among the mediators independent of their absolute levels. In a study by Lisco *et*. *al*., the authors reported distinct clustering of cytokines in semen and blood of HIV infected versus HIV uninfected men, with significantly more associations observed in the infected population. The authors speculated that the multiple associations result in a rigidity in cytokine network and may result in the loss of immune flexibility that is required for efficient functioning of the immune system [47]. In our cohort, Abuse groups showed the most associations and therefore most rigidity, particularly in mediators associated with inflammation. Therefore, it is possible that this level of interdependent engagement can be detrimental in terms of the immune system to respond to pathogens. This concept is speculative at this stage and must be verified by experimental data.

One interesting observation in the heat-maps was the distinct patterns of strong positive associations among growth factors in Depression and Abuse groups compared to Controls. Recent studies indicate potential involvement of growth factors, along with inflammatory cytokines with major depressive symptoms [26,64]. Further, our Abuse+Depression groups also reported increased substance abuse, alcohol consumption and smoking compared to Controls (Table 1), which has been reported by multiple studies to cause general immune dysfunction and impair wound healing pathways (reviewed [14]). Mechanisms involving growth factors in Abuse and Depression in the FRT has not been previously shown and needs to be further elucidated.

Another finding in the present study was that the extent of significance in changes in some of the immune mediators varied depending on whether we adjusted for total protein content. However, CVL total protein content was not significantly different among our groups (Table 2A and 2B). In previous studies, we have reported significantly higher CVL protein content in pregnant women compared to non-pregnant women [44], but not in premenopausal compared to postmenopausal women [39]. Whether the changes in significance that we observe in the present study are biologically relevant is not clear at this point. Further studies are needed to understand whether immune protection in the FRT is altered as a result of these changes.

To our knowledge, this is the first study to characterize a large panel of genital biomarkers in the context of sexual abuse and HIV. Other than the frequently analyzed cytokines/chemokines, we also extended our panel to include anti-HIV/anti-proteases and mediators associated with wound healing. There were several limitations to this study design including small sample sizes, use of extreme phenotypes, and the use of a samples from a pre-existing cohort. Small sample sizes limit the statistical power of the observations, which limits the ability to see small but statistical differences that might exist between groups. Extreme phenotypes were selected in order to maximize potential differences between groups, but these selection criteria also limited the pool of potential samples. Although we selected our Control groups to have no recent abuse of any type, we were not able to control for any non-sexual abuse in their past. Finally, use of samples from an existing cohort means that we could not control for a number of parameters or have control over recorded demographic data. In spite of these limitations and strict criteria of selection of extreme phenotypes, our data does point to immune alterations in FRT of women exposed to chronic sexual abuse. Additional studies with larger sample sizes are warranted to understand the underlying mechanisms further.

## References

[1] Li Y, Marshall CM, Rees HC, Nunez A, Ezeanolue EE, Ehiri JE. Intimate partner violence and HIV infection among women: a systematic review and meta-analysis. J Int AIDS Soc 2014; 17: 18845 doi: 10.7448/IAS.17.1.18845 24560342 10.7448/IAS.17.1.18845 PMC3925800

[2] Lawrence E, Orengo-Aguayo R, Langer A, Brock RL. The impact and consequences of partner abuse on partners. Partner Abuse 2012; 3: 406–428.

[3] Draughon JE. Sexual assault injuries and increased risk of HIV transmission. Adv Emerg Nurs J 2012; 34: 82–87. doi: 10.1097/TME.0b013e3182439e1a 22313905 10.1097/TME.0b013e3182439e1a PMC3522423

[4] World Health Organization. Violence against women: intimate partner and sexual violence against women: intimate partner and sexual violence have serious short-and long-term physical, mental and sexual and reproductive health problems for survivors: fact sheet. 2014.

[5] Women U. Facts and Figures: HIV and AIDS 2015.

[6] Centers for Disease Control and Prevention. Intersection of intimate partner violence and HIV in women. Atlanta, GA: Author.Retrieved from http://www.cdc.gov/violenceprevention/pdf/ipv/13_243567_green_aag-a.pdf 2014.

[7] Iqbal SM, Ball TB, Levinson P, Maranan L, Jaoko W, Wachihi C et al Elevated elafin/trappin-2 in the female genital tract is associated with protection against HIV acquisition. AIDS 2009; 23: 1669–1677. doi: 10.1097/QAD.0b013e32832ea643 19553806 10.1097/QAD.0b013e32832ea643

[8] Brawner BM, Sommers MS, Moore K, Aka-James R, Zink T, Brown KM et al Exploring Genitoanal Injury and HIV Risk Among Women: Menstrual Phase, Hormonal Birth Control, and Injury Frequency and Prevalence. J Acquir Immune Defic Syndr 2016; 71: 207–212. doi: 10.1097/QAI.0000000000000824 26334741 10.1097/QAI.0000000000000824 PMC4712081

[9] Porter KA, Turpin J, Begg L, Brown G, Chakhtoura N, Church E et al Understanding the Intersection of Young Age, Mucosal Injury, and HIV Susceptibility. AIDS Res Hum Retroviruses 2016; 32: 1149–1158. doi: 10.1089/aid.2016.0206 27726428 10.1089/aid.2016.0206 PMC6445180

[10] Klot JF, Auerbach JD, Veronese F, Brown G, Pei A, Wira CR et al Greentree white paper: sexual violence, genitoanal injury, and HIV: priorities for research, policy, and practice. AIDS Res Hum Retroviruses 2012; 28: 1379–1388. doi: 10.1089/AID.2012.0273 22953712 10.1089/aid.2012.0273 PMC3485903

[11] Shah JM, Omar E, Pai DR, Sood S. Cellular events and biomarkers of wound healing. Indian J Plast Surg 2012; 45: 220–228. doi: 10.4103/0970-0358.101282 23162220 10.4103/0970-0358.101282 PMC3495371

[12] Werner S, Grose R. Regulation of wound healing by growth factors and cytokines. Physiol Rev 2003; 83: 835–870. doi: 10.1152/physrev.2003.83.3.835 12843410 10.1152/physrev.2003.83.3.835

[13] Nazli A, Chan O, Dobson-Belaire WN, Ouellet M, Tremblay MJ, Gray-Owen SD et al Exposure to HIV-1 directly impairs mucosal epithelial barrier integrity allowing microbial translocation. PLoS Pathog 2010; 6: e1000852 doi: 10.1371/journal.ppat.1000852 20386714 10.1371/journal.ppat.1000852 PMC2851733

[14] Guo S, Dipietro LA. Factors affecting wound healing. J Dent Res 2010; 89: 219–229. doi: 10.1177/0022034509359125 20139336 10.1177/0022034509359125 PMC2903966

[15] Engeland CG, Sabzehei B, Marucha PT. Sex hormones and mucosal wound healing. Brain Behav Immun 2009; 23: 629–635. doi: 10.1016/j.bbi.2008.12.001 19111925 10.1016/j.bbi.2008.12.001 PMC2746088

[16] Engeland CG, Bosch JA, Cacioppo JT, Marucha PT. Mucosal wound healing: the roles of age and sex. Archives of surgery 2006; 141: 1193–1197. doi: 10.1001/archsurg.141.12.1193 17178961 10.1001/archsurg.141.12.1193

[17] Moller B, Rasmussen C, Lindblom B, Olovsson M. Expression of the angiogenic growth factors VEGF, FGF-2, EGF and their receptors in normal human endometrium during the menstrual cycle. Mol Hum Reprod 2001; 7: 65–72 11134362. 11134362 10.1093/molehr/7.1.65

[18] Ashcroft GS, Jeong MJ, Ashworth JJ, Hardman M, Jin W, Moutsopoulos N et al Tumor necrosis factor-alpha (TNF-alpha) is a therapeutic target for impaired cutaneous wound healing. Wound Repair Regen 2012; 20: 38–49. doi: 10.1111/j.1524-475X.2011.00748.x 22151742 10.1111/j.1524-475X.2011.00748.x PMC3287056

[19] van Bergen BH, Andriessen MP, Spruijt KI, van de Kerkhof PC, Schalkwijk J. Expression of SKALP/elafin during wound healing in human skin. Arch Dermatol Res 1996; 288: 458–462 8844125. 8844125 10.1007/BF02505235

[20] Williams SE, Brown TI, Roghanian A, Sallenave JM. SLPI and elafin: one glove, many fingers. Clin Sci (Lond) 2006; 110: 21–35. 16336202 10.1042/CS20050115

[21] Paiardini M, Muller-Trutwin M. HIV-associated chronic immune activation. Immunol Rev 2013; 254: 78–101. doi: 10.1111/imr.12079 23772616 10.1111/imr.12079 PMC3729961

[22] Devries KM, Mak JY, Bacchus LJ, Child JC, Falder G, Petzold M et al Intimate partner violence and incident depressive symptoms and suicide attempts: a systematic review of longitudinal studies. PLoS Med 2013; 10: e1001439 doi: 10.1371/journal.pmed.1001439 23671407 10.1371/journal.pmed.1001439 PMC3646718

[23] Beydoun HA, Beydoun MA, Kaufman JS, Lo B, Zonderman AB. Intimate partner violence against adult women and its association with major depressive disorder, depressive symptoms and postpartum depression: a systematic review and meta-analysis. Soc Sci Med 2012; 75: 959–975. doi: 10.1016/j.socscimed.2012.04.025 22694991 10.1016/j.socscimed.2012.04.025 PMC3537499

[24] Lorenz T, van Anders S. Interactions of sexual activity, gender, and depression with immunity. J Sex Med 2014; 11: 966–979. doi: 10.1111/jsm.12111 23448297 10.1111/jsm.12111 PMC4410362

[25] Blume J, Douglas SD, Evans DL. Immune suppression and immune activation in depression. Brain Behav Immun 2011; 25: 221–229. doi: 10.1016/j.bbi.2010.10.008 20955778 10.1016/j.bbi.2010.10.008 PMC3025086

[26] Audet MC, Anisman H. Interplay between pro-inflammatory cytokines and growth factors in depressive illnesses. Front Cell Neurosci 2013; 7: 68 doi: 10.3389/fncel.2013.00068 23675319 10.3389/fncel.2013.00068 PMC3650474

[27] Kappelmann N, Lewis G, Dantzer R, Jones PB, Khandaker GM. Antidepressant activity of anti-cytokine treatment: a systematic review and meta-analysis of clinical trials of chronic inflammatory conditions. Mol Psychiatry 2016. 10.1038/mp.2016.167 PMC5794896 27752078

[28] Postal M, Appenzeller S. The importance of cytokines and autoantibodies in depression. Autoimmunity reviews 2015; 14: 30–35. doi: 10.1016/j.autrev.2014.09.001 25242344 10.1016/j.autrev.2014.09.001

[29] Du YJ, Yang CJ, Li B, Wu X, Lv YB, Jin HL et al Association of pro-inflammatory cytokines, cortisol and depression in patients with chronic obstructive pulmonary disease. Psychoneuroendocrinology 2014; 46: 141–152. doi: 10.1016/j.psyneuen.2014.04.020 24882166 10.1016/j.psyneuen.2014.04.020

[30] Bosch JA, Engeland CG, Cacioppo JT, Marucha PT. Depressive symptoms predict mucosal wound healing. Psychosom Med 2007; 69: 597–605. doi: 10.1097/PSY.0b013e318148c682 17766687 10.1097/PSY.0b013e318148c682

[31] Das A. Psychosocial distress and inflammation: Which way does causality flow? Soc Sci Med 2016; 170: 1–8. doi: 10.1016/j.socscimed.2016.10.001 27728857 10.1016/j.socscimed.2016.10.001

[32] Barkan SE, Melnick SL, Preston-Martin S, Weber K, Kalish LA, Miotti P et al The Women's Interagency HIV Study. WIHS Collaborative Study Group. Epidemiology 1998; 9: 117–125 9504278. 9504278

[33] Bacon MC, von Wyl V, Alden C, Sharp G, Robison E, Hessol N et al The Women's Interagency HIV Study: an observational cohort brings clinical sciences to the bench. Clin Diagn Lab Immunol 2005; 12: 1013–1019. doi: 10.1128/CDLI.12.9.1013-1019.2005 16148165 10.1128/CDLI.12.9.1013-1019.2005 PMC1235804

[34] Hessol NA, Weber KM, Holman S, Robison E, Goparaju L, Alden CB et al Retention and attendance of women enrolled in a large prospective study of HIV-1 in the United States. J Womens Health (Larchmt) 2009; 18: 1627–1637 19788344. 19788344 10.1089/jwh.2008.1337 PMC2825719

[35] Radolff L. The CES-D Scale: A self-report depression scale for research in the general population. Applied Psychological Measurement 1977; 1: 385–401.

[36] Radloff LS. The CES-D scale: A self-report depression scale for research in the general population. Applied psychological measurement 1977; 1: 385–401.

[37] Center for Epidemiological Studies-Depression American Psychological Association.

[38] Ochsenbauer C, Edmonds TG, Ding H, Keele BF, Decker J, Salazar MG et al Generation of transmitted/founder HIV-1 infectious molecular clones and characterization of their replication capacity in CD4 T lymphocytes and monocyte-derived macrophages. J Virol 2012; 86: 2715–2728. doi: 10.1128/JVI.06157-11 22190722 10.1128/JVI.06157-11 PMC3302286

[39] Jais M, Younes N, Chapman S, Cu-Uvin S, Ghosh M. Reduced levels of genital tract immune biomarkers in postmenopausal women: implications for HIV acquisition. Am J Obstet Gynecol 2016 27026477. 10.1016/j.ajog.2016.03.041 27026477

[40] Protocol for Neutralizing Antibody Screening Assay for HIV-1 in TZM-bl Cells D. Montefiori. 2010 http://www.hiv.lanl.gov/content/nab-reference-strains/html/Protocol-for-Neutralizing-Antibody-Screening-Assay-for-HIV-1-in-TZM-bl-Cells-November-2010.pdf.

[41] Polis CB, Curtis KM. Use of hormonal contraceptives and HIV acquisition in women: a systematic review of the epidemiological evidence. Lancet Infect Dis 2013; 13: 797–808. doi: 10.1016/S1473-3099(13)70155-5 23871397 10.1016/S1473-3099(13)70155-5

[42] Mirmonsef P, Krass L, Landay A, Spear GT. The role of bacterial vaginosis and trichomonas in HIV transmission across the female genital tract. Curr HIV Res 2012; 10: 202–210 22384839. 22384839 10.2174/157016212800618165 PMC3788616

[43] Kyongo JK, Jespers V, Goovaerts O, Michiels J, Menten J, Fichorova RN et al Searching for lower female genital tract soluble and cellular biomarkers: defining levels and predictors in a cohort of healthy Caucasian women. PLoS One 2012; 7: e43951 doi: 10.1371/journal.pone.0043951 22952818 10.1371/journal.pone.0043951 PMC3432048

[44] Anderson BL, Ghosh M, Raker C, Fahey J, Song Y, Rouse DJ et al In vitro anti-HIV-1 activity in cervicovaginal secretions from pregnant and nonpregnant women. Am J Obstet Gynecol 2012; 207: 65.e1–65.10 22727351. 22727351 10.1016/j.ajog.2012.04.029 PMC3383647

[45] Ghosh M, Fahey JV, Shen Z, Lahey T, Cu-Uvin S, Wu Z et al Anti-HIV activity in cervical-vaginal secretions from HIV-positive and -negative women correlate with innate antimicrobial levels and IgG antibodies. PLoS One 2010; 5: e11366 doi: 10.1371/journal.pone.0011366 20614007 10.1371/journal.pone.0011366 PMC2894072

[46] Lahey T, Ghosh M, Fahey JV, Shen Z, Mukura LR, Song Y et al Selective impact of HIV disease progression on the innate immune system in the human female reproductive tract. PLoS One 2012; 7: e38100 doi: 10.1371/journal.pone.0038100 22675510 10.1371/journal.pone.0038100 PMC3366961

[47] Lisco A, Introini A, Munawwar A, Vanpouille C, Grivel JC, Blank P et al HIV-1 imposes rigidity on blood and semen cytokine networks. Am J Reprod Immunol 2012; 68: 515–521. doi: 10.1111/aji.12015 23006048 10.1111/aji.12015 PMC3493688

[48] Singh PK, Tack BF, McCray PB Jr, Welsh MJ. Synergistic and additive killing by antimicrobial factors found in human airway surface liquid. Am J Physiol Lung Cell Mol Physiol 2000; 279: L799–805. doi: 10.1152/ajplung.2000.279.5.L799 11053013 10.1152/ajplung.2000.279.5.L799

[49] Ong PY, Ohtake T, Brandt C, Strickland I, Boguniewicz M, Ganz T et al Endogenous antimicrobial peptides and skin infections in atopic dermatitis. N Engl J Med 2002; 347: 1151–1160. doi: 10.1056/NEJMoa021481 12374875 10.1056/NEJMoa021481

[50] Wikby A, Ferguson F, Forsey R, Thompson J, Strindhall J, Lofgren S et al An immune risk phenotype, cognitive impairment, and survival in very late life: impact of allostatic load in Swedish octogenarian and nonagenarian humans. J Gerontol A Biol Sci Med Sci 2005; 60: 556–565 15972602. 15972602 10.1093/gerona/60.5.556

[51] Wikby A, Nilsson BO, Forsey R, Thompson J, Strindhall J, Lofgren S et al The immune risk phenotype is associated with IL-6 in the terminal decline stage: findings from the Swedish NONA immune longitudinal study of very late life functioning. Mech Ageing Dev 2006; 127: 695–704. doi: 10.1016/j.mad.2006.04.003 16750842 10.1016/j.mad.2006.04.003

[52] Lajoie J, Mwangi L, Fowke KR. Preventing HIV infection without targeting the virus: how reducing HIV target cells at the genital tract is a new approach to HIV prevention. AIDS research and therapy 2017; 14: 46 doi: 10.1186/s12981-017-0166-7 28893304 10.1186/s12981-017-0166-7 PMC5594430

[53] Krejner A, Grzela T. Modulation of matrix metalloproteinases MMP-2 and MMP-9 activity by hydrofiber-foam hybrid dressing—relevant support in the treatment of chronic wounds. Cent Eur J Immunol 2015; 40: 391–394. doi: 10.5114/ceji.2015.54605 26648787 10.5114/ceji.2015.54605 PMC4655393

[54] Rogers JH, Odoyo-June E, Jaoko W, Bailey RC. Time to complete wound healing in HIV-positive and HIV-negative men following medical male circumcision in Kisumu, Kenya: a prospective cohort study. PLoS One 2013; 8: e61725 doi: 10.1371/journal.pone.0061725 23613918 10.1371/journal.pone.0061725 PMC3626701

[55] Werner S, Grose R. Regulation of wound healing by growth factors and cytokines. Physiol Rev 2003; 83: 835–870. doi: 10.1152/physrev.2003.83.3.835 12843410 10.1152/physrev.2003.83.3.835

[56] Passmore JS, Jaspan HB, Masson L. Genital inflammation, immune activation and risk of sexual HIV acquisition. Curr Opin HIV AIDS 2015 26628324. 10.1097/COH.0000000000000232 PMC6194860 26628324

[57] Herold BC, Keller MJ, Shi Q, Hoover DR, Carpenter CA, Huber A et al Plasma and mucosal HIV viral loads are associated with genital tract inflammation in HIV-infected women. J Acquir Immune Defic Syndr 2013; 63: 485–493. doi: 10.1097/QAI.0b013e3182961cfc 23591635 10.1097/QAI.0b013e3182961cfc PMC3706034

[58] Blish CA, McClelland RS, Richardson BA, Jaoko W, Mandaliya K, Baeten JM et al Genital Inflammation Predicts HIV-1 Shedding Independent of Plasma Viral Load and Systemic Inflammation. J Acquir Immune Defic Syndr 2012; 61: 436–440. doi: 10.1097/QAI.0b013e31826c2edd 22878424 10.1097/QAI.0b013e31826c2edd PMC3494808

[59] Lajoie J, Boily-Larouche G, Juno J, Kirwan S, Fowke K. Mucosal Immune Regulation: Does it Help Thwart HIV. J Clin Cell Immunol S 2012; 7: 2.

[60] Lajoie J, Poudrier J, Massinga Loembe M, Guedou F, Leblond F, Labbe AC et al Chemokine expression patterns in the systemic and genital tract compartments are associated with HIV-1 infection in women from Benin. J Clin Immunol 2010; 30: 90–98. doi: 10.1007/s10875-009-9343-3 19898927 10.1007/s10875-009-9343-3

[61] Singh UP, Singh NP, Murphy EA, Price RL, Fayad R, Nagarkatti M et al Chemokine and cytokine levels in inflammatory bowel disease patients. Cytokine 2016; 77: 44–49. doi: 10.1016/j.cyto.2015.10.008 26520877 10.1016/j.cyto.2015.10.008 PMC4666758

[62] Grimm MC, Elsbury SK, Pavli P, Doe WF. Enhanced expression and production of monocyte chemoattractant protein-1 in inflammatory bowel disease mucosa. J Leukoc Biol 1996; 59: 804–812 8691064. 8691064 10.1002/jlb.59.6.804

[63] Ghosh M, Shen Z, Schaefer TM, Fahey JV, Gupta P, Wira CR. CCL20/MIP3alpha is a novel anti-HIV-1 molecule of the human female reproductive tract. Am J Reprod Immunol 2009; 62: 60–71. doi: 10.1111/j.1600-0897.2009.00713.x 19527233 10.1111/j.1600-0897.2009.00713.x PMC3837341

[64] Kappelmann N, Lewis G, Dantzer R, Jones PB, Khandaker GM. Antidepressant activity of anti-cytokine treatment: a systematic review and meta-analysis of clinical trials of chronic inflammatory conditions. Mol Psychiatry 2016 27752078. 10.1038/mp.2016.167 PMC5794896 27752078

